# Processed EEG Monitoring During Pediatric Procedural Sedation: Correlation Between the Narcotrend Index and the Pediatric Sedation State Scale

**DOI:** 10.1002/pan.70245

**Published:** 2026-06-16

**Authors:** Hendryk Schneider, Daniel Matheisl, Martin Kuntz, Hans Fuchs

**Affiliations:** ^1^ Division of Neonatology and Pediatric Intensive Care Medicine Center for Pediatrics, Medical Center ‐ University of Freiburg, Faculty of Medicine, University of Freiburg Freiburg Germany

**Keywords:** Children, Narcotrend Index, Procedural sedation monitoring, Processed EEG

## Abstract

**Background:**

Objective assessment of sedation depth in children undergoing procedural sedation while maintaining spontaneous breathing and protective reflexes can be challenging. Processed electroencephalogram (pEEG) monitoring systems such as the Narcotrend monitor may provide continuous information regarding the depth of sedation. The aim of this study was to investigate the correlation between the Narcotrend Index (NI) and the Pediatric Sedation State Scale (PSSS) during procedural sedation with propofol and fentanyl in children.

**Methods:**

Children and adolescents aged 6 months to 17 years undergoing procedural sedation with propofol and fentanyl in the procedure room of a pediatric intensive care unit were included. Sedation depth was assessed using the Narcotrend monitor and the Pediatric Sedation State Scale. Spearman's rank correlation coefficient was calculated to evaluate the relationship between the NI and PSSS.

**Results:**

A total of 311 paired measurements (NI and PSSS) from 29 patients were analyzed. Spearman's rank correlation analysis demonstrated a moderate to good correlation between the NI and PSSS (*r* = 0.61; 95% CI 0.54–0.68; *p* < 0.0001). The median NI corresponding to the desired deep sedation level (PSSS 2) was 39 (IQR 30–50), with a 95% confidence interval for the median of 36–42.

**Conclusion:**

The Narcotrend Index demonstrated an acceptable correlation with the Pediatric Sedation State Scale. Processed EEG monitoring using the Narcotrend Index may therefore represent a useful adjunct to clinical monitoring for avoiding both over‐sedation and inadequate sedation in children, particularly for less experienced sedation providers.

**Trial Registration:**

German Clinical Trials Register: DRKS00033129

## Summary

- What is already known about the topic?
  ○ Assessment of sedation depth in children relies primarily on clinical sedation scales, which provide intermittent and potentially stimulus‐dependent evaluations, while processed EEG monitoring offers continuous assessment but remains insufficiently studied in pediatric procedural sedation.
- What new information does this study add?
  ○ This study shows that the Narcotrend Index correlates with a validated clinical sedation scale during propofol–fentanyl procedural sedation in children, supporting its use as an objective adjunct to improve sedation titration and enhance patient safety.

## 1 Introduction

Procedural sedation is frequently required for diagnostic and therapeutic interventions in pediatric patients. The targeted depth of sedation depends on several factors, including the type and duration of the procedure, anticipated pain levels, and the expertise of the sedation provider [1, 2]. However, the intended level of sedation does not always correspond to the sedation depth that is actually achieved. Consequently, objective monitoring of sedation depth may help to improve patient safety and reduce the risk of adverse events.

Clinical sedation scales are widely used to assess the level of sedation in children undergoing diagnostic or therapeutic procedures [1, 3]. The Pediatric Sedation State Scale (PSSS) is a validated instrument that has been used in multiple prospective studies evaluating pediatric sedation and analgesia protocols [3, 4, 5]. Nevertheless, clinical sedation scores provide only intermittent assessments and typically require stimulation of the patient, which may lead to transient arousal.

Processed electroencephalogram (pEEG) monitoring is commonly used in adult patients to guide anesthesia and sedation depth, although data in pediatric populations remain limited. In adults, EEG‐based monitoring has been shown to improve control of anesthetic depth and reduce the risk of inadequate anesthesia [6, 7]. Furthermore, pEEG monitoring has been proposed as a potential objective and non‐disruptive method for assessing sedation depth in children receiving propofol‐based procedural sedation [8]. Continuous analysis of electrocortical activity may therefore provide uninterrupted information regarding sedation depth without requiring stimulation of the patient.

Data on processed EEG monitoring during pediatric procedural sedation remain limited, particularly in the pediatric intensive care unit setting.

The aim of this study was to investigate the correlation between the Narcotrend Index and the PSSS in children undergoing procedural sedation in the procedure room of a tertiary pediatric intensive care unit. In addition, we sought to identify the NI range that most closely corresponds to a moderate to deep sedation state (PSSS level 2).

## 2 Methods

This retrospective observational study included pediatric patients undergoing procedural sedation, which was performed in a dedicated procedure room of our tertiary pediatric intensive care unit.

Procedural sedation was performed by a single dedicated physician (H.S.) with extensive experience in pediatric sedation and intensive care medicine (board‐certified pediatrician with subspecialty training in neonatology, pediatric intensive care, and pediatric cardiology, and additional training in anesthesia, with more than 14 years of clinical experience and several hundred procedural and critical care sedations).

Patients were eligible if they were classified as American Society of Anesthesiologists (ASA) physical status I or II, were aged between 6 months and 17 years, and had no contraindications to procedural sedation. Patients with significant neurological disease or those receiving chronic medication affecting the central nervous system were excluded.

All patients were admitted from the general pediatric ward and were not receiving any sedative or opioid medication prior to the procedural sedation. Sedative agents (propofol and fentanyl) were administered exclusively as part of the standardized procedural sedation protocol.

Sedation was performed according to our unit's protocol using a single dose of fentanyl citrate (1–1.5 μg/kg), followed by repeated doses of propofol 1% (0.5–1 mg/kg) until the desired sedation depth was achieved. All patients received supplemental oxygen via nasal cannula at flow rates between 0.5 and 4 L/min throughout the sedation. Vital signs were continuously monitored according to the unit protocol.

Sedation was assessed every 1–2 min using the PSSS (Table 1) by a designated single investigator (H.S.) who was blinded to the Narcotrend monitor.

**TABLE 1:** The Pediatric Sedation State Scale (PSSS) – also see [3].

State	Behavior
5	*Patient is moving* (*purposefully or nonpurposefully*) in a manner that impedes the proceduralist and *requires forceful immobilization*. This includes crying or shouting during the procedure, but vocalization is not required. Score is based on movement
4	*Moving* during the procedure (awake or sedated) that *requires gentle immobilization* for positioning. May verbalize some discomfort or stress, but there is no crying or shouting that expresses stress or objection
3	*Expression of pain or anxiety* on face (may verbalize discomfort), but *not moving* or impeding completion of the procedure. May require help positioning (as with a lumbar puncture) but does not require restraint to stop movement during the procedure
2	*Quiet (asleep or awake), not moving* during procedure, and no frown (or brow furrow) indicating pain or anxiety. No verbalization of any complaint
1	*Deeply asleep with normal vital signs*, but requiring *airway intervention* and/or assistance (e.g., central or obstructive apnea, etc.)
0	*Sedation associated with abnormal physiologic parameters* that require acute intervention (i.e., oxygen saturation < 90%, blood pressure is 30% lower than baseline, bradycardia receiving therapy)

Patients were monitored using a Narcotrend system (Narcotrend‐Compact M, Narcotrend, Hannover, Germany), as already published [8]. After skin preparation, EEG electrodes were placed on the left and right lateral forehead with the maximal achievable distance. A reference electrode was placed on the mid‐forehead, and electrode impedance was maintained below 6 kΩ.

Due to limited patient acceptance, particularly in younger or anxious children, EEG electrodes were applied after the onset of sedation. To ensure a standardized procedure across all age groups and avoid variability related to differing levels of cooperation, electrode placement was not attempted prior to sedation.

EEG signals were processed using proprietary software (Version 3.10, Narcotrend), generating a numerical Narcotrend Index representing the depth of sedation [9].

In accordance with standard safety recommendations [1] procedural sedation and the procedure itself were performed by different physicians.

Local anesthesia was used for most procedures (e.g., surgical interventions, catheter procedures, biopsies) as part of standard clinical practice, with the exception of endoscopic procedures and joint aspirations.

The Narcotrend Index values corresponding with the predominant EEG features and clinical state of alertness are illustrated in Table 2.

**TABLE 2:** Narcotrend Index, corresponding predominant electroencephalogram (EEG) features and clinical description (also see [8]).

Narcotrend index	Clinical description	Predominant EEG characteristics
100–95	Alertness	α‐waves
94–90		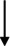
89–85	Tiredness/light sedation	β‐waves
84–80		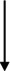
79–75		
74–70	Sedation/light anesthesia	θ‐waves
69–65		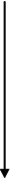
64–57		
56‐47	Deep sedation/general anesthesia	
46–37		
36–27		
26–20	General anesthesia with deep hypnosis	δ‐waves
19–13		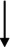
12–5		Burst suppression
4–0	Very deep general anesthesia	Isoelectric EEG

Spearman's rank correlation coefficient was calculated to evaluate the relationship between the NI and PSSS scores using GraphPad Prism (GraphPad Prism V. 9.5, GraphPad Software, USA). A two‐tailed *p*‐value of < 0.05 was considered statistically significant.

The study was approved by the institutional review board of the University of Freiburg (Research Ethics Committee Reference Number 24‐1232‐S1‐retro, July 16th 2024).

## 3 Results

Twenty‐nine patients undergoing procedural sedation between November 1st, 2022 and March 31st, 2023 were included in the analysis (Table 3), with characteristics summarized accordingly. A total of 311 paired measurements of Narcotrend Index values and corresponding PSSS scores were obtained.

**TABLE 3:** Patient characteristics (*n* = 29).

Characteristic	Value
Age (years), median (IQR)	7 (2.5–11.5)
Gender (female/male)	15/14
Weight (kg), median (IQR)	22 (13.3–43.5)
Duration of sedation (min), median (IQR)	35 (30–50)
Fentanyl dose (μg/kg), median (IQR)	1.1 (1.0–1.4)
Total propofol dose (mg/kg), median (IQR)	6.8 (4.9–8.4)
*Procedures*	
Surgical procedures (removal of implanted central venous catheter, abdominal drainage, wound treatment)	11
Bone marrow puncture, lumbar puncture	6
Esophagogastroduodenoscopy	3
Central venous catheter placement	3
Joint aspiration	3
Liver‐ or kidney biopsy	3

**FIGURE 1:**
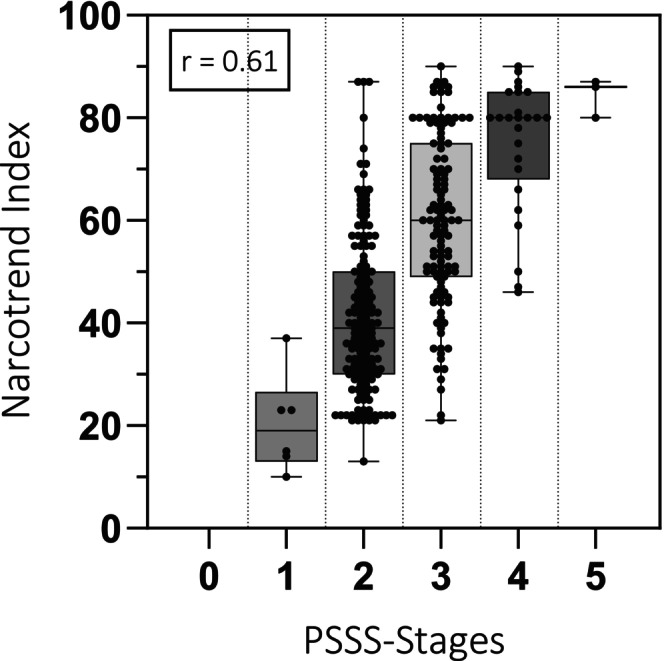
Box and whisker plots (maximum, upper quartile value, median value, lower quartile value, minimum) with individual patient data of Narcotrend Index (NI) displayed as a scatter overlay versus Pediatric Sedation State Scale (PSSS) and the Spearman rank correlation coefficient (*r*).

Spearman's rank correlation analysis demonstrated a moderate to good correlation between NI and PSSS (*r* = 0.61; 95% CI 0.54–0.68; *p* < 0.0001). The distribution of NI values across different PSSS levels showed that deeper levels of sedation were generally associated with lower NI values (Figure 1).

For the target sedation level of PSSS 2, the median NI value was 39 (IQR 30–50), with a 95% confidence interval of 36–42 (also see Table 4).

**TABLE 4:** Descriptive statistics displaying the NI (Narcotrend Index) values for the 5 different documented PSSS (Pediatric Sedation State Scale) levels.

PSSS	1	2	3	4	5
Number of NI values	6	163	114	25	3
Minimum NI	10.00	13.00	21.00	46.00	80.00
25% Percentile NI	13.00	30.00	49.00	68.00	80.00
Median NI	19.00	39.00	60.00	80.00	86.00
75% Percentile NI	26.50	50.00	75.00	85.00	87.00
Maximum NI	37.00	87.00	90.00	90.00	87.00
Range	27.00	74.00	69.00	44.00	7.000
95% CI of median					
Actual confidence level	96.88%	95.86%	95.13%	95.67%	75.00%
Lower confidence limit for NI	10.00	36.00	55.00	72.00	80.00
Upper confidence limit for NI	37.00	42.00	63.00	81.00	87.00
Mean NI	20.33	41.28	59.87	74.92	84.33
Std. Deviation	9.668	15.06	16.49	12.90	3.786
Std. Error of Mean	3.947	1.180	1.545	2.581	2.186

## 4 Discussion

This study investigated the relationship between the processed EEG‐derived Narcotrend Index and the PSSS in children undergoing procedural sedation. Our findings demonstrate a moderate to good correlation between the two measures.

The median NI value associated with the desired sedation level (PSSS 2) was approximately 39 (IQR 30–50) and the confidence interval for the median was 36–42 (also see Table 4). This finding is consistent with previous work by Weber et al. [8], where the median NI associated with the targeted UMSS level of 4 (“unarousable”) was 38 (with a 95% confidence interval for the median of 36–43). Furthermore, in a recent publication of Bruns et al. investigating the correlation of the Comfort Scale and the Narcotrend Index, the median NI value for the targeted Comfort Scale for deep sedation (Comfort Scale 11–14) was 44 (IQR 32–63) [10]. Comparing these results, it is reasonable to say that for propofol guided sedation in children (age 6 months to 18 years), the target range for the pEEG derived Narcotrend Index is probably around the value of 40.

However, a considerably wide range of NI values (especially in the deeper sedation levels) as well as overlap across different PSSS levels was observed. Similar variability has been reported in other studies and may reflect individual differences in EEG patterns during sedation.

These findings highlight an important limitation of processed EEG monitoring in pediatric procedural sedation. Similar NI values may correspond to a broad range of clinical sedation states, indicating that NI alone is not sufficiently reliable to guide sedation depth.

Importantly, reliance on NI values without concurrent clinical assessment could potentially result in inappropriate titration of sedative agents. In particular, attempts to achieve lower NI targets may increase the risk of over‐sedation and associated adverse events such as airway compromise. Therefore, the Narcotrend Index should be considered strictly as an adjunct to, and not a replacement for, clinical evaluation using validated sedation scales.

Our findings support the use of processed EEG monitoring as a complementary tool that may enhance situational awareness, as an adjunct to clinical monitoring, but must always be interpreted within the clinical context.

Sedation medications may also influence EEG patterns and therefore affect pEEG‐derived indices. For example, midazolam premedication has been shown to produce distinct EEG changes in adult patients [11]. For a sedation protocol for children undergoing gastrointestinal endoscopy with remifentanil plus propofol, a target range for the NI of 65 ± 5 was defined [12], which is notably higher than the target range value we observed with fentanyl and propofol. Differences in sedation protocols may therefore partially explain variations in NI target ranges reported in the literature.

Age‐related maturation of EEG patterns represents another important factor influencing the reliability of processed EEG monitoring in children [7, 13]. For this reason, infants younger than 6 months were not included in our study. The feasibility in this age group seems to be limited because of the lower probability of indicating a Narcotrend Index [14]. Moreover, age‐related differences in EEG characteristics and propofol pharmacodynamics may additionally influence processed EEG indices in pediatric patients. Although our cohort included a wide age range (6 months to 17 years), we did not perform age‐stratified analyses due to the limited sample size, which would have resulted in small and statistically unreliable subgroups. Furthermore, propofol dosing during procedural sedation in our cohort showed relatively limited variability (median 6.8 mg/kg, IQR 4.9–8.4), reflecting our pragmatic titration approach.

We believe that one strength of our observational study is the use of the 6‐point PSSS. This sedation scale may have the advantage of discriminating more optimally, especially for the state of over‐sedation (state 0 and 1), when acute interventions for correcting abnormal physiological parameters or airway assistance are necessary, which should be avoided during procedural sedation. Contrary, the deepest UMSS sedation level 4 describes an unarousable patient but is not able to discriminate between deep sedation with preserved protective reflexes and the state of over‐sedation with abnormal vital signs. In our opinion, this seems to be a major advantage compared to the UMSS. In the 29 procedural sedations we investigated, we did not experience any major adverse events. The PSSS state 0 was never protocolled.

Multiple measurements were obtained from individual patients during the same sedation episode. However, due to variability in procedure duration and measurement frequency, we were not able to identify consistent intra‐individual patterns of Narcotrend Index values over time. This heterogeneity limited the ability to construct comparable time‐course analyses across patients.

The evaluation of individual NI trajectories during sedation may represent a promising approach for personalized sedation management, but would require prospective studies with standardized measurement intervals and larger sample sizes.

The relatively small sample size and the low number of observations at very deep sedation levels limit the ability to define clinically relevant threshold values or to draw conclusions regarding the prevention of over‐sedation. Therefore, our findings should be interpreted as exploratory and hypothesis‐generating.

While our study demonstrates a correlation between NI and PSSS, evidence regarding the impact of processed EEG monitoring on clinical outcomes is limited. However, previous work, such as the randomized controlled trial by Weber et al. [12], suggests that EEG‐guided sedation may reduce drug consumption and recovery time, as well as episodes of oversedation in selected pediatric populations.

### 4.1 Limitations

Several limitations should be considered. First, the number of included patients was relatively small and the procedures performed were heterogeneous with regard to invasiveness and duration. However, this pragmatic design reflects routine clinical practice in pediatric procedural sedation.

Second, the analysis was retrospective and derived from a single center—we introduced the Narcotrend monitoring primarily for clinical familiarization, wanted to verify the reliability of this monitoring in our clinical setting, and make the data available.

Third, baseline (pre‐sedation) Narcotrend values were not obtained, as electrode placement prior to sedation was not feasible in many patients due to limited cooperation. This may limit the interpretation of absolute index changes.

Moreover, potential age‐dependent effects represent an important limitation and should be addressed in future studies with larger sample sizes.

The use of local anesthesia in most procedures may have reduced nociceptive stimulation and thereby influenced the relationship between clinical sedation scores and EEG‐derived indices. Reduced behavioral responses due to effective analgesia could potentially result in lower PSSS levels despite relatively higher Narcotrend Index values. This factor should be considered when interpreting the correlation between NI and PSSS.

Finally, multiple measurements were obtained from individual patients during the same sedation episode, which may introduce clustering effects. However, the primary aim of this study was to explore the overall relationship between Narcotrend Index values and a clinical sedation score across routine clinical practice.

## 5 Conclusions

In this study, the Narcotrend Index demonstrated a moderate to good correlation with the PSSS in children aged 6 months to 17 years undergoing procedural sedation with propofol and fentanyl.

Our findings suggest that an NI value of approximately 40 may correspond to an adequate level of deep sedation.

Nevertheless, Narcotrend (and other pEEG) monitoring should only be considered a supplementary tool and should always be interpreted in conjunction with the necessary clinical evaluation.

Summarized, continuous EEG‐derived monitoring may be useful as an adjunct to clinical assessment during pediatric procedural sedation. However, given the variability of Narcotrend Index values across sedation levels and the limited sample size of this study, it should not be used as a standalone tool for guiding sedation depth.

## Funding

The authors have nothing to report.

## Ethics Statement

This study was approved by the institutional review board of the University of Freiburg (Research Ethics Committee Reference Number 24‐1232‐S1‐retro, July 16th 2024).

## Conflicts of Interest

The authors declare no conflicts of interest.

## Data Availability

The datasets used and analyzed during this study are available from the corresponding author upon request.
